# The Physics of Life and Quantum Complex Matter: A Case of Cross-Fertilization

**DOI:** 10.3390/life1010003

**Published:** 2011-09-29

**Authors:** Nicola Poccia, Antonio Bianconi

**Affiliations:** Department of Physics, Sapienza University of Rome, P. le A. Moro 2, 00185 Roma, Italy

**Keywords:** complexity, criticality, life, photosynthesis, high temperature superconductivity, Fano shape resonance

## Abstract

Progress in the science of complexity, from the Big Bang to the coming of humankind, from chemistry and biology to geosciences and medicine, and from materials engineering to energy sciences, is leading to a shift of paradigm in the physical sciences. The focus is on the understanding of the non-equilibrium process in fine tuned systems. Quantum complex materials such as high temperature superconductors and living matter are both non-equilibrium and fine tuned systems. These topics have been subbjects of scientific discussion in the Rome Symposium on the “Quantum Physics of Living Matter”.

Today we have reached a fantastic knowledge basis on the complexity of the living cell. We know now nearly all molecular components of the cell and their interactions with the use of synchrotron radiation, lasers, scanning atomic force microscopes, nuclear magnetic resonance techniques, and other physical methods joined with the advanced capability of computers for data storage and analysis.

While the classification of biological components and processes is well known, the physics of the matter in the living state remains elusive. Quantum physics is generally neglected in classical biophysics courses and in biophysical research. In the first ten years of this century, new experimental and theoretical results have provided evidence for the role of quantum mechanics in living matter [1,2,3,4,5,6]. These works are attracting a growing scientific interest and have been published in highly ranked science journals indicating that they start to be considered as a viable roadmap in the search for the new physics of living matter

To boost research in this field and to bring together the few active scientists in this field, a Symposium on “Quantum Physics of Living Matter” has been organized in July 2011 in Italy at Rome University “La Sapienza” [7]. The Symposium has been organized together with the conference on “Quantum Phenomena in Complex Matter 2011” focussing on superconductivity at high temperature in complex systems to create new links between the two scientific communities.

The matter of debate has been on the non-trivial quantum effects that appear in the macroscopic world of the living cell. The presentations have focused first on the large-amplitude dynamical conformational deviations in the DNA from the DNA canonical Watson-Crick double-helix template presented as the main talk of Alan R. Bishop. The lattice deviations in DNA have a great similarity with the local deviations from average structures that have been identified and assigned functionality over the last decade in a great variety of organic and inorganic non-biological materials. This is a topic of great interest for the community of scientists interested in striped lattice textures in high temperature superconductors. The presented effective nonlinear model used to analyze and simulate the strand separation (bubble) dynamics of certain promoter DNA sequences have taken advantage of these concepts developed in seemingly different fields [8,9,10,11].

The second topic covered by the symposium has been on the intrinsic wave nature of biological molecules. M. Arndt has presented a series of recent experiments carried out in Vienna targeted at generating spatial superposition of isolated organic molecules [12], showing that under suitable circumstances even a single rather complex object may exist in two or more positions at the same time. Since complex objects are also present in biological membranes, the quantum phenomena in photosynthesis have been deeply discussed. It has been recently discovered that, contrary to expectations of physicists as well as biologists, the energy transport during photosynthesis, from the chlorophyll pigment that captures the photon to the reaction centre where glucose is synthesized from carbon dioxide and water, is highly coherent even at ambient temperature and in the cellular environment [13,14]. It has been proposed that the light harvesting antennae function by propagation of excitons through dipole–dipole interactions, and accumulation of energy at the reaction centre.

The theoretical interpretation based on a coupled oscillator model implementing wave dynamics has been presented by Apoorva D. Patel [15]. He has presented the spatial search algorithm with nearest neighbor coupling and a reflection oracle applied to photosynthesis for the study of the dynamics for concentrating the energy of the system at the target location, analogous to the trapping mechanism of a resonating cavity. Recent empirical findings concerning an illumination-driven transition in the biomolecular membrane architecture of the purple bacteria Rsp. Photometricum have been interpreted by Felipe Caycedo with a model that allows exploring the possibilities for photon-chemical conversion due to excitations transfer in the biomolecular membrane beyond classical hopping dynamics [16]. New quantum algorithms for the simulation of the protein folding have been presented by Pietro Faccioli, that sensibly reduce the time of computation. In particular, the Dominant Reaction Pathways (DRP) approach has been shown to be useful to study quantum delocalization effects in the conformational transitions of a peptide [17].

A shape resonance mechanism for evading decoherence effects at high temperature has been proposed to be active in the processes of molecular association and dissociation in living matter [18,19] such as in high temperature superconductors that show scale free structural organization [20] and a complex time evolution [21] making these complex materials that show high temperature superconductivity like living systems in a complex far from equilibrium state.

Seth Lloyd from MIT has proposed that the convergence of timescales in photosynthesis can be understood as an example of the “quantum Goldilocks effect”. This means that the natural selection tends to drive quantum systems to the degree of complexity that is ‘just right’ for attaining maximum efficiency [22]. He has discussed how the convergence of time scales is hard to establish since the convergence of time scales makes quantum systems hard to model. Since the convergence of time scales can then either assist energy transport, or interfere with it in naturally occurring systems that have undergone a long process of dynamic refinement via natural selection, the convergent processes typically help each other out.

Recently the Lloyd group has developed a non-perturbative, non Markovian master equation technique for simulating the behavior of complex quantum systems over a wide variety of time d energy scales [23] and it has been applied to the Fenna-Matthews-Olson complex (FMO), a seven-chromophore energy transport complex in green sulphur bacteria [24]. It has been found that the convergence of timescales in FMO is tuned to give high transport efficiency (virtually 100%) that is robust over several decades of variation in the underlying parameters of the system.

Convergence of the dark energy scale with the energy scale of membrane potential has been also proposed to have had a role in the refinement of biological machinery during evolution and in the determination of the onset of life in the Universe [25].

In conclusion, we hope that this conference will mark a point for scientists focussing on statistical physics far from equilibrium and development of new quantum mechanics ideas that could play a key role in the foundations of a physics of living matter. We also believe that this conference has given a first input for cross fertilization between the fields of high temperature superconductivity and quantum physics of living matter.
